# Supplementary material for: Transfection of hPSC-Cardiomyocytes Using Viafect™ Transfection Reagent
Source: Methods Protoc. 2020 Aug 9;3(3):57. doi: 10.3390/mps3030057 (PMC7564709; doi:10.3390/mps3030057)
Supplement: Supplementary file 1 [file mps-03-00057-s001.zip › mps-872332-supplementary-proofed/mps-872332-supplementary-for proof_proofread.docx]

**Supplementary Data**

Technical Note

Transfection of hPSC-Cardiomyocytes using Viafect™ Transfection Reagent

Sara E Bodbin *, Chris Denning * and Diogo Mosqueira *

Division of Cancer & Stem Cells, Biodiscovery Institute, University of Nottingham, NG7 2RD Nottingham, UK

***** Correspondence: sara.bodbin1@nottingham.ac.uk (S.E.B.); chris.denning@nottingham.ac.uk (C.D.); Diogo.Mosqueira@nottingham.ac.uk (D.M.)

1. Supplementary Methods

1.1. hPSC Derivation and Differentiation

Human pluripotent stem cells (hPSCs) were cultured in Matrigel (MT)-coated vessels in Essential 8 medium and differentiated in a monolayer protocol as previously described [1]. The REBL-PAT hiPSC line was derived from a skin punch biopsy from a male subject, and transduced using CytoTune-iPS Sendai Reprogramming (Thermofisher #A16517). iPSC colonies were then isolated by manual dissection. Additionally, male HUES7 were utilised as a source of hESC-cardiomyocytes [2]. These cell lines were used between passages 20–50 (all patient skin biopsies were donated via informed consent under the approval of Research Ethics Committee—number 09/H0408/74). hPSC-cardiomyocytes (hPSC-CMs) at over ~90% purity were dissociated at day 20 of culture, by collagenase treatment as previously described [3] and replated as indicated in the Methods section of the manuscript.

1.2. Brain Natriuretic Peptide (BNP) Assay

BNP assay was performed as previously described [4]. In brief, hPSC-CMs were transfected as described in the Methods section of the manuscript, and the medium was replaced by RPMI+B27 without serum, on the day after transfection and every other day thereafter. On the day of the assay, the cells were incubated with 1μg/ml Brefeldin A (Sigma #B7651) for 3 h at 37 °C and 5% CO_2_. Thereafter, the cells were fixed and immunostained as described below. Image acquisition was performed as described below and BNP signal intensity was determined in the perinuclear region of the cardiomyocytes.

1.3. Immunocytochemistry (ICC) and High Content Imaging

The hPSC-CMs were washed with PBS and fixed in 4% paraformaldehyde (PFA, Sigma) at RT for 15 min. Afterwards, the cells were washed in 0.1% Tween-20 (Fisher Scientific) in PBS, permeabilized with 0.1% Triton-X (Sigma) in PBS for 15 min at RT, and incubated with 4% goat serum (Sigma) in PBS (blocking solution) for 1 h at RT, to prevent unspecific antibody binding. Subsequently, primary antibody incubation was performed overnight at 4 °C in blocking solution, at the following dilutions: anti-α-actinin-1:800 (Sigma #A7811), anti-TroponinT-1:500 (Abcam #45932), anti-ProBNP4-1:500 (Abcam #13115). Thereafter, the samples were washed three times with 0.1% Tween-20 in PBS and incubated with Alexa Fluor secondary antibodies (Life Technologies) at 1:400 in blocking solution for 1h at RT. Afterwards, the cells were washed with 0.1% Tween-20 in PBS for (3x 5min), followed by nuclei and/or whole-cell counterstaining with 0.5 µg/ml DAPI (Sigma #D9542) or Cell Mask (1:10000, Invitrogen #H32721) in PBS, respectively, for 30 min at RT. Samples were subsequently washed and stored at 4 °C in PBS until automated image acquisition was performed in the Operetta high-content imaging system (Perkin Elmer), and analysed using Harmony high-content imaging analysis software, using the algorithms reported in [4] for the determination of cardiomyocyte purity and BNP expression.

2. Supplementary Figures


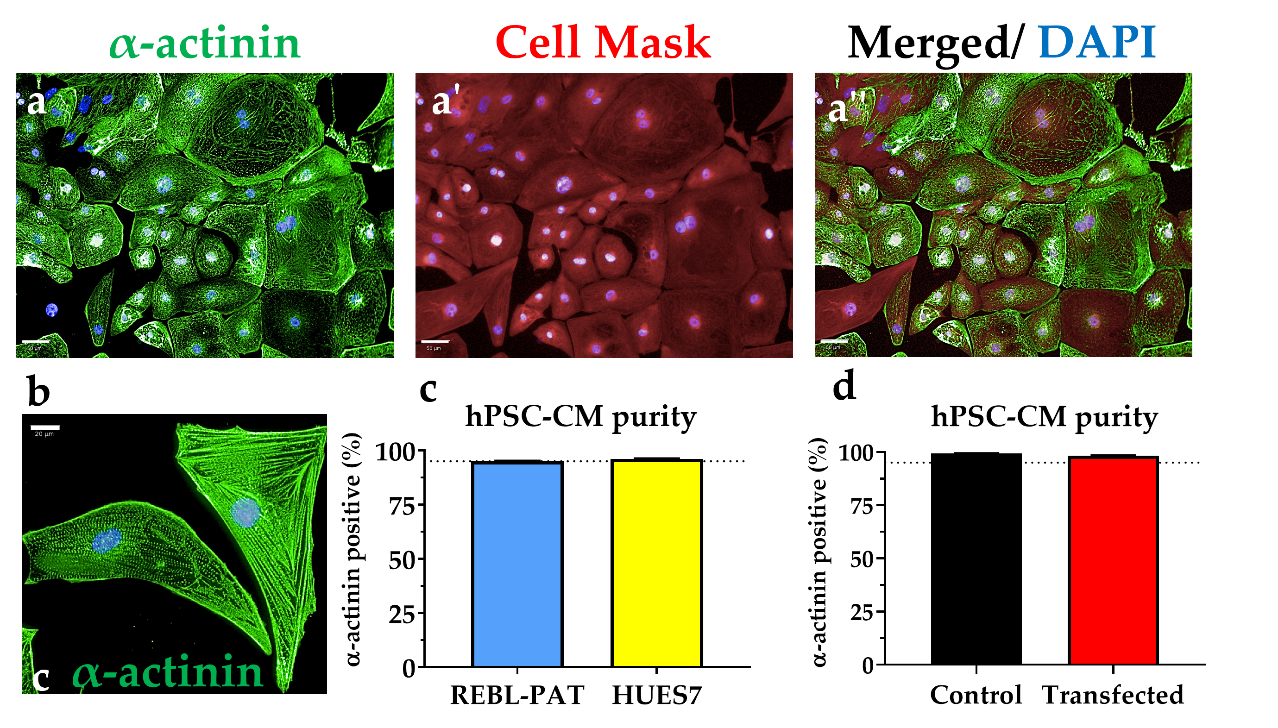


**Supplementary Figure S1**—**Determination of the purity of** **human pluripotent stem cell- cardiomyocytes (hPSC-CMs) used in this study**. **a–a’’)** A high-content imaging method was used to quantify the percentage of cells (counterstained by a cell mask) expressing the cardiomyocyte marker α-actinin, labelled by immunostaining (scale bar = 50 um). **b)** Sarcomeric banding exhibited by the cardiomyocytes immunostained for alpha-actinin (scale bar = 20 um). **c)** Purity of cardiomyocytes resulting from the cardiac differentiation of hPSC-CMs is typically ≥ 95% (dotted line). **d)** hPSC-CMs retain high purities upon transfection with Viafect. Data: mean ±SEM, N=5 biological replicates performed across 8 wells.


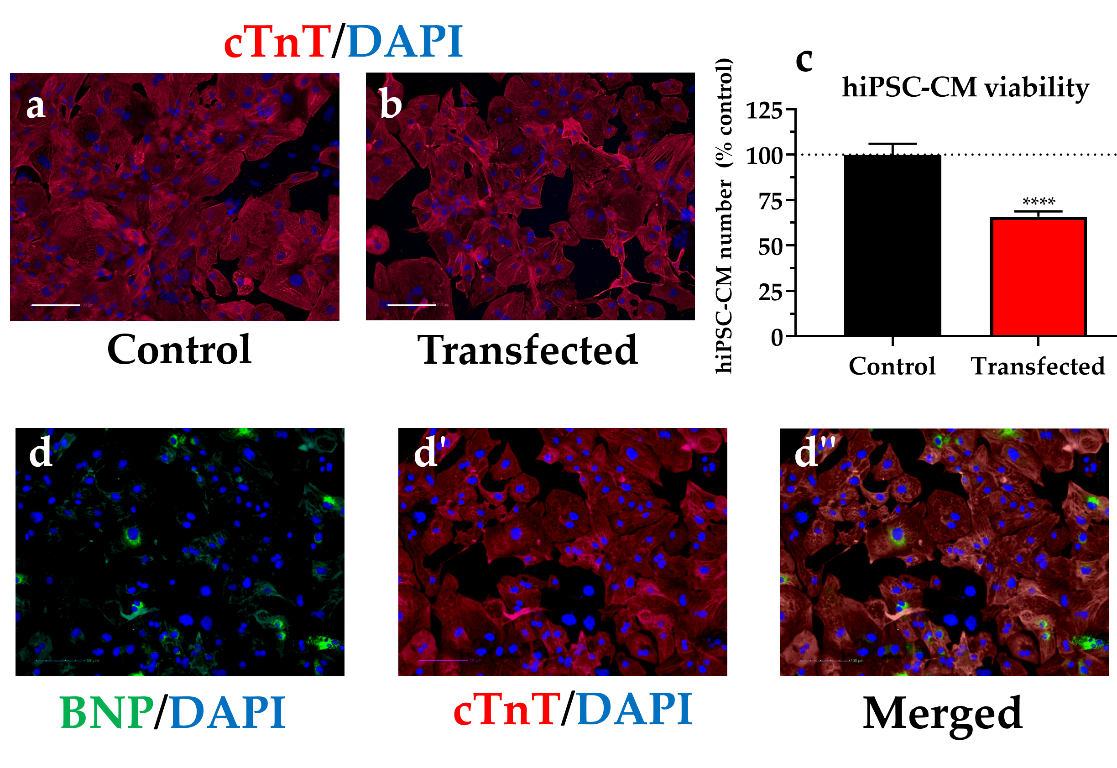


**Supplementary Figure S2*—*****Effects of transfection on hiPSC-CM viability and phenotypic assays. a–c)** Transfected hiPSC-CMs showed a ~34% reduction in cell numbers relative to the untransfected controls, indicating a ~66% cell viability, which still ensured compatibility with the phenotypic assays such as the **d–d’’)** identification of hypertrophic marker brain natriuretic peptide (BNP) in the perinuclear region of cardiomyocytes. ****p<0.001, unpaired Student’s t test. Data: mean ±SEM, N=5 biological replicates performed across 8 wells (scale bar = 100 μm)*;* cTnT – cardiac Troponin T.

References

1. Mosqueira, D.; Mannhardt, I.; Bhagwan, J.R.; Lis-Slimak, K.; Katili, P.; Scott, E.; Hassan, M.; Prondzynski, M.; Harmer, S.C.; Tinker, A., et al. CRISPR/Cas9 editing in human pluripotent stem cell-cardiomyocytes highlights arrhythmias, hypocontractility, and energy depletion as potential therapeutic targets for hypertrophic cardiomyopathy. *Eur. Heart J.* **2018**, *39*, 3879–3892.

2. Cowan , C.A.; Klimanskaya , I.; McMahon , J.; Atienza , J.; Witmyer , J.; Zucker , J.P.; Wang , S.; Morton , C.C.; McMahon , A.P.; Powers , D., et al. Derivation of Embryonic Stem-Cell Lines from Human Blastocysts. *N. Engl. J. Med.* **2004**, *350*, 1353–1356.

3. Breckwoldt, K.; Letuffe-Breniere, D.; Mannhardt, I.; Schulze, T.; Ulmer, B.; Werner, T.; Benzin, A.; Klampe, B.; Reinsch, M.C.; Laufer, S., et al. Differentiation of cardiomyocytes and generation of human engineered heart tissue. *Nat. Protocols* **2017**, *12*, 1177–1197.

4. Mosqueira, D.; Lis-Slimak, K.; Denning, C. High-Throughput Phenotyping Toolkit for Characterizing Cellular Models of Hypertrophic Cardiomyopathy In Vitro. *Methods Protoc.* **2019**, *2*.
